# The Role and Therapeutic Value of Syndecan-1 in Cancer Metastasis and Drug Resistance

**DOI:** 10.3389/fcell.2021.784983

**Published:** 2022-01-18

**Authors:** Sen Guo, XinYi Wu, Ting Lei, Rui Zhong, YiRan Wang, Liang Zhang, QingYi Zhao, Yan Huang, Yin Shi, Luyi Wu

**Affiliations:** ^1^ Shanghai University of Traditional Chinese Medicine, Shanghai, China; ^2^ Key Laboratory of Acupuncture and Immunological Effects, Shanghai Research Institute of Acupuncture and Meridian, Shanghai, China; ^3^ Department of Acupuncture and Moxibustion, Yueyang Hospital of Integrated Traditional Chinese and Western Medicine Affiliated to Shanghai University of Traditional Chinese Medicine, Shanghai, China; ^4^ Outpatient Department, Shanghai Research Institute of Acupuncture and Meridian, Shanghai, China

**Keywords:** syndecan-1, metastasis, drug resistance, therapy, cancer

## Abstract

Metastasis and relapse are major causes of cancer-related fatalities. The elucidation of relevant pathomechanisms and adoption of appropriate countermeasures are thus crucial for the development of clinical strategies that inhibit malignancy progression as well as metastasis. An integral component of the extracellular matrix, the type 1 transmembrane glycoprotein syndecan-1 (SDC-1) binds cytokines and growth factors involved in tumor microenvironment modulation. Alterations in its localization have been implicated in both cancer metastasis and drug resistance. In this review, available data regarding the structural characteristics, shedding process, and nuclear translocation of SDC-1 are detailed with the aim of highlighting strategies directly targeting SDC-1 as well as SDC-1-mediated carcinogenesis.

## Introduction

Cell surface proteoglycans are responsible for many aspects of cellular behavior. The four-member syndecan (SDC) family of heparan sulfate proteoglycans includes SDC-1 (CD138), 2, 3 and 4 (Rhodes and Simons, 2007; Couchman, 2010). Expressed primarily on the surface of epithelial and plasma cells, SDC-1 has been the most intensively studied of the four (Saunders et al., 1989). A key cell surface adhesion molecule, SDC-1 contains heparan sulfate (HS) chains which interact with a large number of molecules crucial in the maintenance of cell morphology and intercellular signaling such as extracellular matrix components, growth factors and integrins. Dysregulation of SDC-1 may promotes carcinogenesis, cancer recurrence and resistance to chemotherapy. Expression of SDC-1 may thus serve as a potential marker to identify patients predisposed to drug-resistant disease or metastasis on initial cancer diagnosis. As the membrane-anchored SDC-1 undergoes both nuclear translocation as well as extracellular shedding, emerging studies have focused on location-specific roles played by this protein in tumor pathology.

Although data detailing the relationship between tumor progression and changes in the location of SDC-1 expression are scarce, analysis of available literature would nevertheless further understanding of molecular events associated with variations in SDC-1 localization. Here, we review the positional variability of this proteoglycan, how tissue-specific metastasis, in turn, is affected, as well as relevant influences on tumor resistance to treatment. In addition, we provide an evidence-based foundation for the development of potential clinical management strategies targeting SDC-1 in the setting of malignancy.

## Structural and Biochemical Characteristics of SDC-1

**FIGURE 1:**
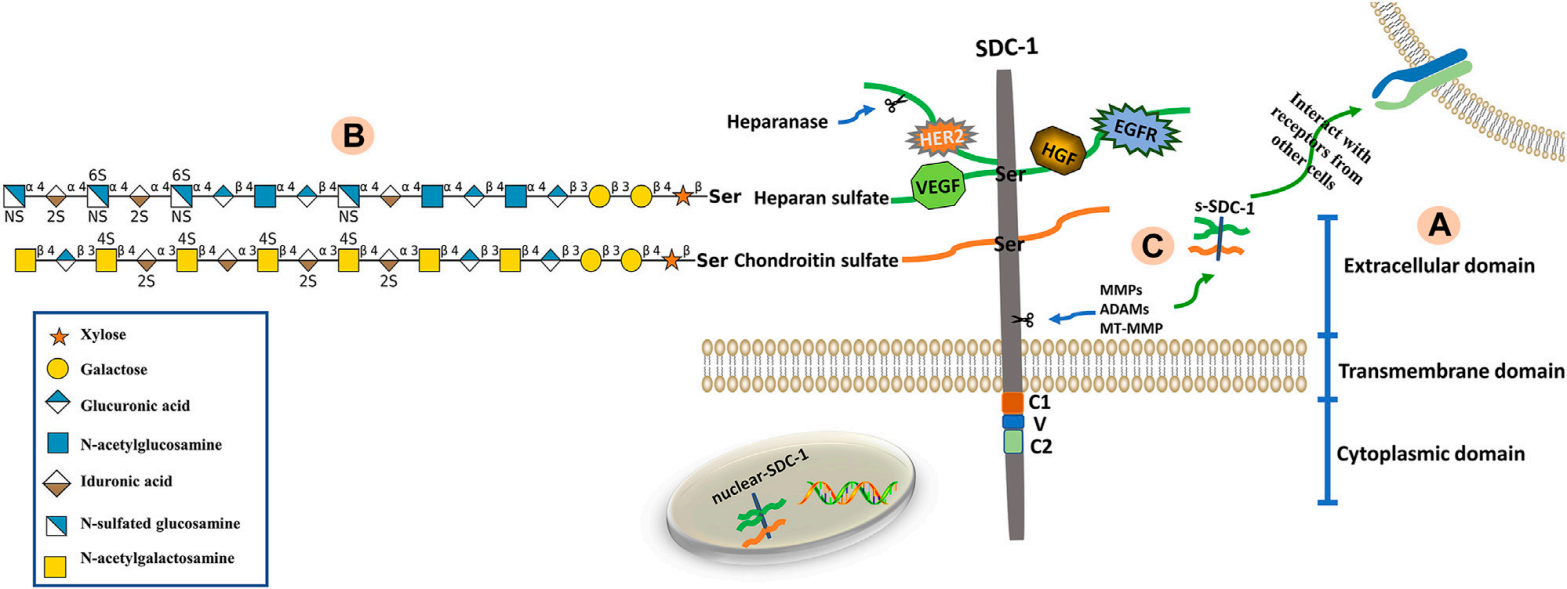
Structure of SDC-1. **(A)** The SDC-1 core protein consists of three major (extracellular, transmembrane and cytoplasmic) domains. **(B)** The extracellular domain is bound *via* glycosaminoglycan (heparan sulfate, HS; chondroitin sulfate, CS) chains. The HS and CS chains are composed of repeating disaccharide units (glycan structures are represented according to the Symbol Nomenclature for Glycans (SNFG) (Cheng et al., 2017)) linked to core protein serine residues. Growth factors and receptors (e.g., HER2, VEGF, HGF and EGFR) bind to HS chains, which can be fragmented by heparanase. **(C)** The extracellular domain of SDC-1 is cleaved by sheddases (e.g., MMPs, ADAMs, MT-MMP), a phenomenon known as shedding. This results in the release of attached glycosaminoglycan chains as well as any bound ligands from the cell surface into the extracellular environment.

Syndecans are heparan sulfate proteoglycans with core proteins possessing heparan sulfate chains. The SDC-1 core protein can also be modified by chondroitin sulfate (CS) chains (Jenkins et al., 2018). The core proteins of syndecans are composed of three domains; namely extracellular (ectodomain, ED), transmembrane (TMD) and cytoplasmic (CD) domains (Bernfield et al., 1999). The short, highly-conserved CD can be further divided into conserved C1 (membrane-proximal) and C2 (membrane-distal) regions that flank a V region. The V region sequence is distinct among each of the four syndecan family member proteins and likely confers unique functional characteristics (Tkachenko et al., 2005; De Rossi and Whiteford, 2013). The C1 region interacts with actin-bound proteins and participates in endocytosis, while the C2 region interacts with a variety of PDZ proteins, such as syntenin, and thus functions in exosome formation and cytoplasmic trafficking (Das et al., 2003; Maday et al., 2008; Jung et al., 2016). The V region is critical for lamellipodial extension, actin bundling and cell migration (Chakravarti et al., 2005). Maintenance of the actin cytoskeleton and membrane trafficking are primarily regulated by the CD (Alexopoulou et al., 2007). The ED is composed of two or three consecutive Ser-Gly sequences surrounded by hydrophobic and acidic residues that serve as HS or CS attachment sites (Bourdon et al., 1987). The HS chains consist of unbranched, repeating disaccharide units of either glucuronic (GlcUA) or iduronic (IdoA) acid alternating with unsubstituted or N-substituted glucosamine, on which the substituents are either acetate (GlcNAc; N-acetylglucosamine) or sulfate (GlcNS; N-sulfated glucosamine) (Zhang et al., 1995; Park, 2016). The CS chains, which are closer to the membrane surface are synthesized onto a linkage tetrasaccharide (GlcUA-Gal-Gal-Xyl), covalently bound to core protein serine residues *via* the alternating addition of GalNAc and GlcUA units by CS synthases (Ogawa et al., 2010). A defining characteristic of these polysaccharides is the presence of sulfate and uronic acid residues, which endow them with significant anionic properties. As such, numerous proteins containing basic amino acid clusters are capable of interacting with HS chains (Mulloy et al., 2017). Sulfatases such as Sulf1 and Sulf2 change the affinity of HS-binding ligands by removing 6-O-sulfate groups from HS chains (El Masri et al., 2020). The structural composition of SDC-1 is shown in Figure 1.

## Translocation of SDC-1

Changes in SDC-1 location uniquely impact cellular function and encompass anchoring to the cell membrane, ED shedding or nuclear translocation.

### Shedding of the SDC-1 Ectodomain

Syndecans bound to the cell surface act as multifunctional modulators of signaling. As such, proteolytic shedding of the ED, which converts membrane-bound SDC-1 into a shed form, significantly affects signaling functions. Shed SDC-1 retains its HS chains along with bound ligands that endow it with the capacity to act in paracrine or autocrine manners as well as functionality as a competitive inhibitor (Bertrand and Bollmann, 2019). Cell surface receptor dynamics are thus regulated by ED shedding, which results in competition for ligands in the pericellular environment by intact syndecans and potentially eliminates their co-receptor role in various signaling pathways (Piperigkou et al., 2016). Various metalloproteinases including matrix metalloproteinases (MMPs), membrane type MMPs (MT-MMP) and a disintegrin and metalloproteinase with thrombospondin motifs (ADAMTS) are responsible for cleaving SDC-1 from the cell surface (Bode and Maskos, 2003; Gomis-Rüth, 2009; Hadigal et al., 2020). In addition to HS chain cleavage, heparanase promotes SDC-1 shedding *via* the regulation of MMP-9 and urokinase-type plasminogen activator expression (Ramani et al., 2016). The cleavage of HS chains by heparanase not only accelerates the shedding process by providing sheddases access to the SDC-1 core protein, but also allows the extracellular binding of growth factors to the cleaved chains to disseminate across a long distance (Matsuo and Kimura-Yoshida, 2013; Rangarajan et al., 2020). In the conserved CD regions, tyrosine phosphorylation likewise results in ED shedding (Manon-Jensen et al., 2010). Shed SDC-1 proceeds to mediate extracellular signaling in an environment-dependent manner.

### Nuclear Translocation of SDC-1

Intranuclear SDC-1 has been detected in the setting of multiple myeloma (Stewart et al., 2015), prostate cancer (Farfán et al., 2020) and mesothelioma (Kumar-Singh et al., 2020). Co-localization of SDC-1 with tubulin in the mitotic spindle further confirmed this phenomenon (Zong et al., 2009; Szatmári et al., 2017). Nuclear import of protein requires its targeting by nuclear localization signal (NLS) short peptide sequences (Duverger et al., 1995). The minimal sequence required for the tubulin-dependent nuclear translocation of SDC-1 is considered to be the conserved juxtamembrane RMKKK motif present in its CD (Zong et al., 2009; Zong et al., 2010; Zong et al., 2011). Shed SDC-1 has recently been reported to undergo nuclear translocation in both tumor and bone marrow stromal cells, with the presence of HS chains required for this process (Stewart et al., 2015). Intracellular signaling can be altered by the presence of intranuclear SDC-1 *via* protein phosphorylation and post-translational modification. The majority of intranuclear SDC-1 was detected in discrete patches within euchromatin, indicating specific localization to regions of active gene transcription. In the human myeloma cell, intranuclear SDC-1 interacts with the enzyme histone acetyltransferase p300 (HAT) *via* HS chains to decrease its activity and thus histone acetylation (Purushothaman et al., 2011). In aggressive myeloma cells, heparanase mediates the loss of nuclear SDC-1, enhancing HAT activity and resulting in upregulated vascular endothelial growth factor (VEGF) and matrix metalloproteinase-9 (MMP-9) expression (Szatmári et al., 2017; Amin et al., 2020). In addition to mediating the nuclear translocation of SDC-1 and histone H3 acetylation, HS chains that enter the nucleus play important roles in cell signaling. Intranuclear HS chains control transcription by inhibiting DNA topoisomerase; this prevents DNA relaxation and the binding of transcription factors (Kovalszky et al., 1998). Direct inhibition of transcription factors by intranuclear HS chains likely occurs due to their DNA-binding domains containing sequences with a high-affinity for heparan (Dudás et al., 2000; Stewart and Sanderson, 2014). Furthermore, HS can transport heparan-binding growth factors such as hepatocyte growth factor (HGF) and fibroblast growth factor 2 (FGF2) into the nucleus *via* internalization. Adherence to HS chains by ligands, pathogens, peptides and exosomes can also lead to their nuclear importation (Christianson and Belting, 2014). Consequently, cancer cell pathophysiology including tumor growth, metastasis and angiogenesis is uniquely affected by whether SDC-1 is membrane-bound, shed or transported to the cell nucleus.

## Promotion of Metastasis by SDC-1

Cancer cell metastasis is generally divided into five stages: local invasion, intravasation, circulatory system survival, extravasation and colonization. Cellular migration entails loss of focal cellular adhesion, decrease in adherence to cell substrates, formation of new adhesions, and finally, polymerization and depolymerization of the actin cytoskeleton (Mythreye and Blobe, 2009). The precise mechanisms of relevant signal transduction to target cells, however, remain unknown. Due to the variability of the SDC-1 domain, changes in its location have been speculated to correlate with the occurrence of metastasis. Highly expressed on epithelial cells, the heparan-binding domains of SDC-1 are capable of binding laminin, collagen, fibronectin and thrombospondin, resulting in activation of focal adhesion kinase (FAK) signaling. Cell-to-substrate adhesion *via* SDC-1 binding the laminin *α* chain is also facilitated (Salmivirta et al., 1994; Hoffman et al., 1998; Chakravarti et al., 2005; Ogawa et al., 2007). Moreover, fluorescence recovery after photobleaching studies have revealed the TMD to control cell motility and thus adhesion complex protein cycling as well as focal adhesion turnover modulation (Altemeier et al., 2012a). The degradation of extracellular substrates by MT1-MMP as well as its activation of pro-MMP-2 and pro-MMP-13 effectively stimulates matrix turnover and significantly influences malignant metastasis (Ingvarsen et al., 2020). In colorectal carcinoma cells, SDC-1 expression was reported to reduce MMP-9 levels, impede invasion into type I collagen and promote cell adhesion by boosting intercellular cell adhesion molecule-1 (ICAM-1) expression (Wang et al., 2019). Heparanase, however, promotes SDC-1 shedding, which is accompanied by upregulated MMP levels and increased metastasis (Ramani et al., 2016). In addition, SDC-1 regulates focal adhesion dynamics *via* control of Rap1 (a small GTPase that switches integrins to a high-affinity state) to slow cell disadhesion and suppress migration (Boettner and Van Aelst, 2009; Altemeier et al., 2012b). In the setting of various malignancies, expression of the chaperone protein Hsp90 on the cell surface is often upregulated (Birbo et al., 2021). Of note, Hsp90 was found to play a role in tumor cell invasion and metastasis by promoting EGFR3/HER2 dimerization and EGFR signaling (Sidera et al., 2008), thus increasing both MMP and extracellular matrix protein stability and activity (Correia et al., 2013). Extracellular Hsp90 can be bound by HS chains to induce human glioblastoma A-172 and fibrosarcoma HT1080 cell metastasis (Snigireva et al., 2019). In breast cancer (Nadanaka et al., 2021), multiple myeloma (Purushothaman and Sanderson, 2020) and pancreatic cancer (Chen et al., 2020), greater SDC-1 shedding was similarly found to associate with increased metastasis, indicating that shed SDC-1 likely serves as a major facilitator for malignant cellular invasion. Shed SDC-1 may promotes metastasis and invasion *via* growth factors linked to its HS chains. Meanwhile, c-Met, a protein that possesses tyrosine kinase activity, mediates mesenchymal-epithelial interactions (Birchmeier et al., 2003; Nakamura et al., 2011). In myeloma cells, HGF binds to cell surface-bound SDC-1 with the resultant SDC-1/HGF complex stimulating cell migration *via* the c-Met receptor upon SDC-1 cleavage (Ramani et al., 2011). Shed SDC-1 binds VEGF, anchoring it close to the matrix and further promoting endothelial cell invasion (Purushothaman et al., 2010).

Epithelial cells on the invasive front typically acquire migratory and invasive capabilities by overcoming physical barriers during the epithelial-mesenchymal transition (EMT). Nuclear translocation of SDC-1 further enhances its modulation of the EMT as well as tumor invasiveness. Cadherin (isoform) switching is an EMT characteristic and has been linked to the development of invasive and metastatic features in epithelial malignancies (Loh et al., 2019). A positive correlation between Snail expression and nuclear SDC-1 translocation was reported in prostate cancer cells (Farfán et al., 2020); cells overexpressing Snail exhibited increased nuclear SDC-1 levels in comparison with cytoplasmic concentrations (Millanes-Romero et al., 2013). The nuclear translocation of SDC-1, however, was also reported to facilitate elimination of mesenchymal and invasive characteristics among human B6FS fibrosarcoma cells, with loss of nuclear SDC-1 related to cell elongation and E- to N-cadherin switching during the TGF-β1-induced EMT in human A549 lung cancer cells (Kumar-Singh et al., 2021). The influence of intranuclear SDC-1 thus likely differs between different tumors and further study is required to elucidate how nuclear SDC-1 controls the EMT.

**FIGURE 2:**
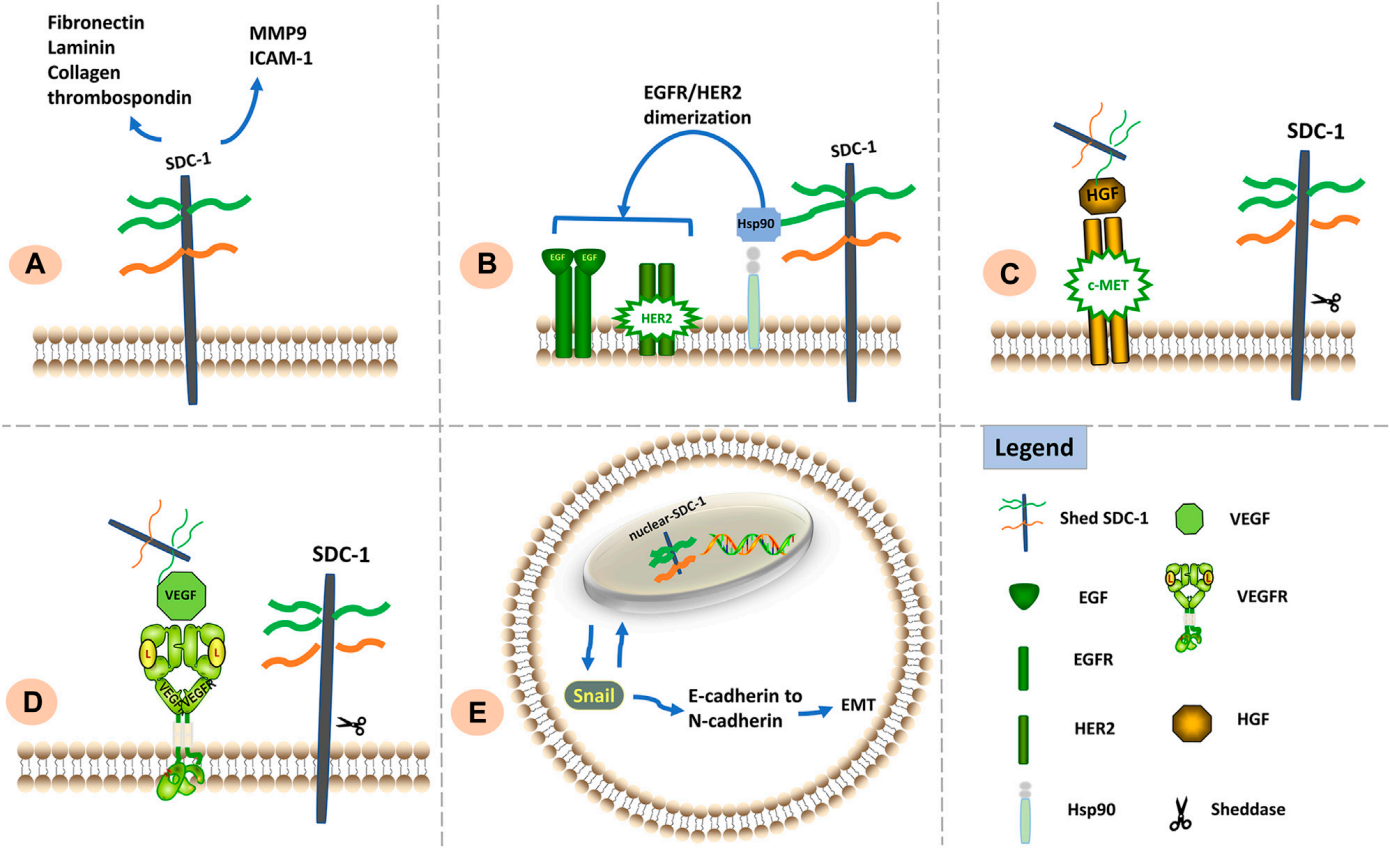
Signaling functions of SDC-1 relevant to cell metastasis and invasion. **(A)** SDC-1 interacts with a vast number of extracellular matrix molecules such as MMP9, ICAM1, fibronectin, laminin, thrombospondin, and collagen; such interactions are facilitated via multivalent binding of these molecules to HS chains, thereby influencing cellular adhesive properties. **(B)** The HS chains of SDC-1 can bind extracellular Hsp90 to promote EGFR/HER2 dimerization and induce metastasis. **(C)**. Shed SDC-1/HGF complex stimulates cell migration via the c-Met receptor. **(D)**. Shed SDC-1 binds VEGF to promote endothelial cell invasion. **(E)**. Nuclear translocation of SDC-1 modulates the EMT via interaction with Snail, resulting in enhanced tumor cell invasiveness.

Effects of SDC-1 differ according to its location: the ED is crucial for maintaining cell adhesion whereas the TMD and CD are important for inhibiting cell migration; cell adhesion and migration capabilities also appear to be influenced by the intranuclear presence of SDC-1 (Zong et al., 2011). Signaling functions of SDC-1 relevant to metastasis and invasion are shown in Figure 2.

## Impact of SDC-1 on Cancer Resistance to Therapy

Shed SDC-1, along with factors that bind to its HS chains, facilitates establishment of a tumor microenvironment that promotes disease recurrence and robust growth *via* the enhancement of growth factor signaling in host cells. As such, it is hypothesized that radiotherapy or chemotherapy may indeed increase extracellular SDC-1 deposition, subsequently leading to tumor recurrence and metastasis (Masola et al., 2014; Bandari et al., 2018).

Treatment with doxorubicin, dexamethasone, cisplatin and carfilzomib was found to significantly increase levels of shed SDC-1 lacking the CD (Ramani and Sanderson, 2014). Furthermore, chemotherapy was found to potentially promote HGF/c-Met/IL-11 activation *via* SDC-1 shedding, exacerbating bone destruction in the setting of myeloma (Ramani et al., 2011). Shed SDC-1 was also reported to promote VEGF signaling, thus increasing the rate of angiogenesis (Jung et al., 2016; Javadi et al., 2020). The HS chains of shed and full-length SDC-1 compete to bind downstream epithelial growth factor receptor (EGFR), subsequently facilitating resistance to chemotherapy in colorectal cancer cells (Wang et al., 2014). Interestingly, higher levels of shed SDC-1 were found to associate with chemoresistance; chemoresistant cells both expressed higher levels of SDC-1 mRNA and, in turn, produced more of the protein (Ramani and Sanderson, 2014). Greater levels of SDC-1 were also found to correlate with reduced responsiveness to cyclophosphamide and epirubicine therapy in cells obtained from pre-chemotherapy biopsies of breast cancer tissue (Götte et al., 2006).

Phosphatidylinositol 3-kinase (PI3K) is a lipid kinase that regulates a variety of cellular processes, while protein kinase B (AKT) is a major downstream effector of PI3K signaling that modulates pathways critical for the inhibition of apoptosis, stimulation of cell growth and modulation of cellular metabolism; aberrant PI3K/AKT activation is thus considered to be as among the significant cause of chemoresistance (Liu et al., 2020). Membrane expression of SDC-1 has likewise been linked to increased chemoresistance in hepatic carcinoma cells *via* PI3K/AKT pathway changes (Yu et al., 2020). Considering that shed SDC-1 promotes the EMT, high levels of shed SDC-1 were found to upregulate expression of EMT-TFs including ZEB1, Snail1 and Snail2 in breast and pancreatic cancer models to induce expression of the stemness factors SOX2, BMI1, and OCT4, thus facilitating chemoresistance (Pradella et al., 2017).

Heparanase upregulation in the setting of anti-myeloma therapy may serve as markers of chemoresistance and eventual relapse. Heparanase was found present within autophagosomes and chemoresistance triggered by heparanase was reported to be partly mediated by enhanced autophagy (Shteingauz et al., 2015). In addition, while increased heparanase activity was reported in lapatinib-resistant HER2 and EGFR-positive breast cancer cells, heparanase inhibition was found to re-sensitize these cells to lapatinib (Zhang et al., 2015).

Exosomes transport proteins, mRNA and miRNA between tumor and host cells; their role in the intercellular shuttling of drug-resistant contents has recently garnered attention (Chen et al., 2014). The SDC-1 CD interacts with syntenin and ALIX to generate a complex that allows intraluminal vesicles to emerge within endosomal membranes, thus contributing to the formation of exosomes (Roucourt et al., 2015). Myeloma cells exposed to elevated heparanase levels exhibited increased secretion of exosomes containing SDC-1 and heparanase (Bandari et al., 2018). As both SDC-1 and heparanase possess tumorigenic properties, exosomes containing them significantly alter both the local microenvironment as well as distant cell functions.

After malignant cells achieve target organ infiltration, they proceed to adapt to their new microenvironment. Tumor cells lacking appropriate signaling functionality either become unable to grow or enter a relatively dormant state (Pantel and Brakenhoff, 2004). Among malignant cells that enter such a quiescent state, metastatic breast cancer cells were noted to escape dormancy after vascular cell adhesion molecule-1(VCAM-1) upregulation. Abnormal VCAM-1 expression was found to induce disseminated tumor cell binding to osteoclasts expressing the *α*4β1 integrin, resulting in bone metastasis (Lu et al., 2011). Considering that shed SDC-1 increases VCAM-1 expression, shed SDC-1 delivered from distant tumor microenvironments similarly promotes growth in otherwise dormant cancer cells and thus facilitates disease relapse and metastasis (de Oliveira Neves et al., 2019).

**FIGURE 3:**
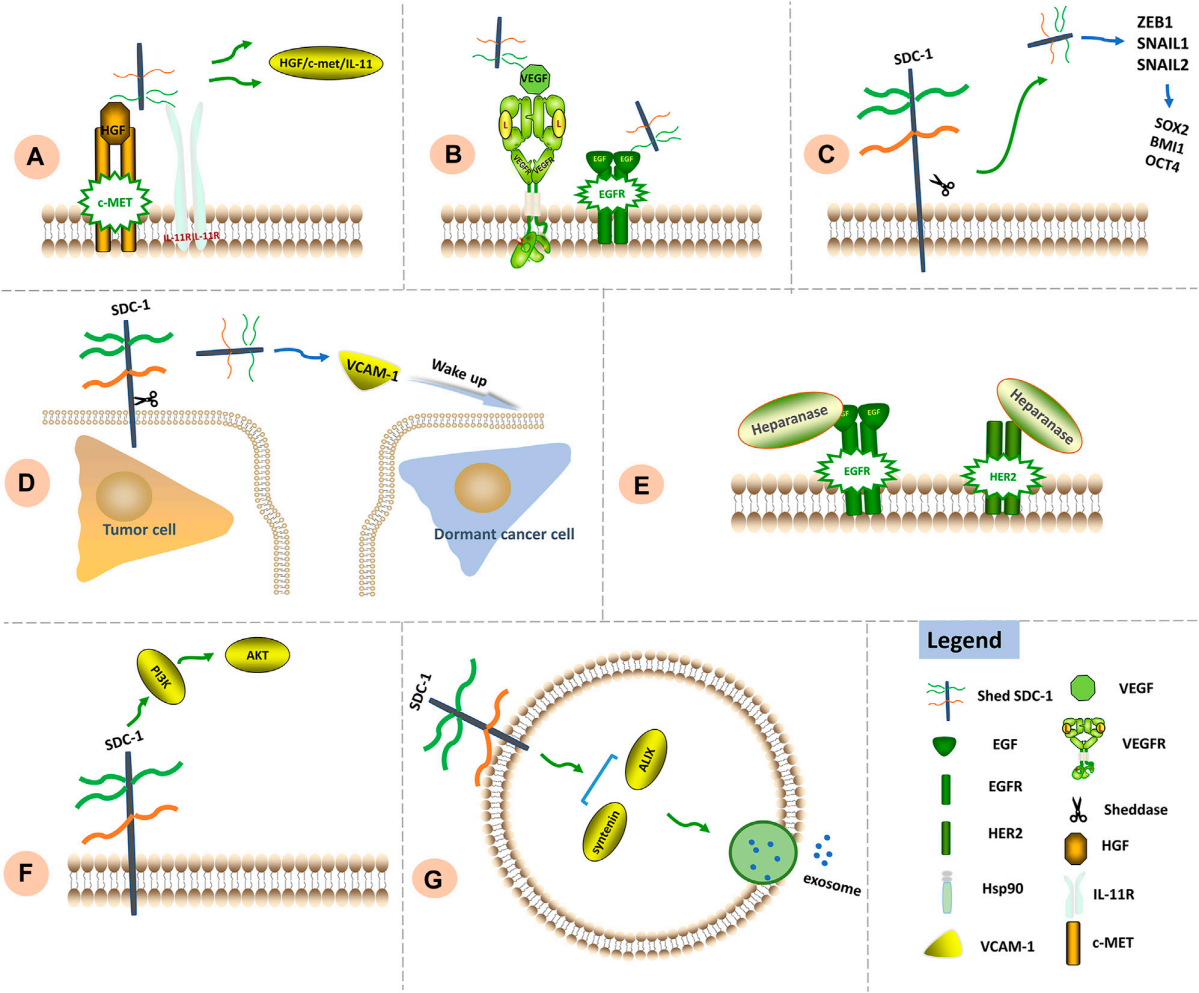
Signaling functions of SDC-1 in chemoresistance. **(A)** Chemotherapy leads to SDC-1 shedding. Shed SDC-1 binds HGF/c-Met/IL-11, exacerbating drug resistance. **(B)** Shed SDC-1 promotes VEGF signaling. The HS chains of shed and full-length SDC-1 compete to bind downstream EGFR, thereby increasing the rate of angiogenesis as well as chemoresistance. **(C)** Shed SDC-1 upregulates EMT-TFs including ZEB1, Snail1 and Snail2 to induce expression of the stemness factors SOX2, BMI1 and OCT4, thus facilitating chemoresistance. **(D)** Shed SDC-1 increases VCAM-1 expression and subsequently promotes growth of otherwise dormant cancer cells, thus facilitating disease relapse and metastasis. **(E)** Increased heparanase activity contributes to lapatinib resistance *via* regulation of HER2 and EGFR signaling. **(F)** The modulation of PI3K/AKT signaling by SDC-1 has been associated with chemoresistance. **(G)** The SDC-1 cytoplasmic region binds syntenin and ALIX to generate a complex that allows intraluminal vesicles to emerge within endosomal membranes, thus facilitating exosome formation.

Combined clinical application of MMP inhibitors for the purpose of impeding SDC-1 shedding along with chemotherapy may offer a unique approach aimed at preventing formation of microenvironments conducive to tumor recurrence. Significant signaling functions of SDC-1 in chemoresistance are shown in Figure 3.

## Perspectives for Therapeutic Intervention Targeting SDC-1

Due to its multiple roles in cancer pathophysiology, SDC-1 is an appealing molecular target for therapeutic strategies. In this section, we summarize progress made regarding the targeting of SDC-1 for therapeutic purposes in the setting of malignancy.

### Targeting of SDC-1

Interaction between SDC-1 and the extracellular matrix plays an essential role in cancer pathogenesis. Treatment with zoledronate significantly downregulates the expression of SDC-1 and integrins ανβ3, ανβ5 and α5β1; this medication is thus considered to be a powerful anti-cancer agent particularly useful for the inhibition of breast cancer cell proliferation, migration and matrix invasion (Dedes et al., 2012). Nimesulide, a non-steroidal anti-inflammatory drug (COX-2 specific inhibitor), blocks the tumorigenic activities of SDC-1 in the setting of primary effusion lymphoma (George Paul et al., 2011). Indatuximab ravtansine (BT062), a monoclonal antibody connected to the cytotoxic agent DM4 (ravtansine), specifically targets cells expressing SDC-1. When absorbed by target cells, the highly-specific DM4 produces cytotoxic effects with minimal systemic toxicity (Schönfeld et al., 2017; Jagannath et al., 2019).

A number of cellular pathways are potential targets for the inhibition of carcinogenic effects exerted by SDC-1. The completely humanized SDC-1 recombinant antibody OC-46F2 reduces SDC-1/VEGFR-2 activity in tumor microenvironments, consequently blocking vascular maturation and tumor growth in the setting of malignant melanoma and experimental models of ovarian cancer (Orecchia et al., 2013). The αVβ3-SDC-1 interaction is likely necessary for FAK activation and the subsequent upregulation of MMP-2 and MMP-9, important steps in tumor metastasis. The combinative polypeptide CBD–HepII inhibits expression of αVβ3 and SDC-1, thus decreasing interactions between these two receptors in B16 melanoma cells and inhibiting pulmonary metastasis of tumor cells in the circulation (Gong et al., 2008). Increased cell migration, invasion and MMP production are positively correlated with the co-localization of β1 integrin and SDC-1 in breast cancer cells (Gong et al., 2008). As such, the prevention of SDC-1 and β1 integrin co-localization is considered to be a potentially effective therapeutic strategy. Synstatin, peptide mimetics of the docking motifs in the syndecans, disrupts interaction among integrins, IGFR1, VEGFR and SDC-1, thereby resulting in significantly decreased angiogenesis and tumorigenesis *in vivo* (Beauvais et al., 2009; Rapraeger, 2013; Rapraeger et al., 2013; Gao et al., 2021).

### Targeting of Shed SDC-1

Batimastat (BB-94), a broad-spectrum MMP inhibitor that also inhibits SDC-1 shedding, reduces ascites and disrupts breast, ovarian and colorectal carcinogenesis (Macaulay et al., 1999). NCS 405020, another small-molecule inhibitor that prevents the homodimerization of MT1-MMP, blocks the activity of this complex *in vivo* and reduces SDC-1 shedding (Remacle et al., 2012). Benzo(*α*)pyrene was found to promote pulmonary carcinogenesis in BALB/C mice *via* increased shedding of SDC-1 from epithelial cells, whereas all-trans retinoic acid (A-TRA) was found to block this process (Ramya et al., 2012). Use of MMP inhibitors and A-TRA has been suggested in conjunction with chemotherapy to avoid the potentially severe side effects of cancer progression or recurrence caused by chemotherapy-induced SDC-1 shedding (Kawano et al., 2013; Ali et al., 2019). Tranexamic acid, another therapy identified as a serine protease inhibitor that prevents SDC-1 shedding, may potentially serve as an inhibitor of metastasis in certain predisposed patients (Diebel et al., 2018). Antithrombin III, a plasma protein with both potent anticoagulant and anti-inflammatory properties, also prevents SDC-1 shedding and thus plays roles in protection of the endothelial barrier and inhibition of metastasis (Lopez et al., 2020).

### Heparan Sulfate-Based Therapy

Heparanase cleaves the HS chains of SDC-1 at certain locations, and heparanase inhibition decreases SDC-1 shedding. Although heparin is a heparanase inhibitor, it is not clinically utilized in cancer therapy due to its anti-coagulant effect. Heparin-combination therapy is developed and widely used (Casu et al., 2007). Use of modified heparin, small molecule inhibitors and function-blocking monoclonal antibodies are other methods of heparanase inhibition (Ramani et al., 2013). For example, SST0001, a modified heparin, significantly reduces *in vivo* heparanase activity and controls levels of growth factors including HGF and VEGF, thereby preventing angiogenesis in human pediatric sarcoma models (Cassinelli et al., 2013). The molecule M402 with a 6 kDa molecular weight differs from SST0001 as it lacks N-acetylation and likely has a broader spectrum of growth factor-binding (Zhou et al., 2011; Kaur et al., 2021). Such heparin mimics effectively disturb the tumor microenvironment and are even more effective when combined with medications that directly target tumor cells. Preclinical studies have revealed that SST0001 in combination with dexamethasone exerts a significant anti-tumor effect in multiple myeloma xenograft mouse models, markedly reducing the subcutaneous growth of different multiple myeloma cell lines (Ritchie et al., 2011). Importantly, as both SDC-1 and heparanase participate in exosome generation, heparanase inhibition reduces tumor growth as well as exosome-derived tumor recurrence (Wu et al., 2021). The inhibition of proteolysis in the ED of SDC-1 is another method that targets SDC-1 shedding. As noted above, commonly used chemotherapy and radiation regimens promote heparanase upregulation and increase SDC-1 shedding in malignancies such as myeloma (Bandari et al., 2018), pancreatic cancer (Ramani and Sanderson, 2014) and medulloblastoma (Asuthkar et al., 2014). The monoclonal antibodies 9E8 and H1023 neutralize heparanase enzymatic activity and prevent spontaneous hepatic metastasis of ESb lymphoma cells from the primary tumor (Weissmann et al., 2016). Ovarian cancer cell proliferation and migration is effectively suppressed by PG545, a completely sulfated synthetic tetrasaccharide with anti-heparanase activity, when treatment is combined with paclitaxel and cisplatin (Winterhoff et al., 2015). Suramin is another small molecule inhibitor that reduces heparanase activity and inhibits FGF-2 and caspase-3 expression (Tayel et al., 2014). Heparanase activity is similarly inhibited by PI-88, an HS-like sulfated oligosaccharide. Preliminary clinical trials of hepatocellular carcinoma patients treated with PI-88 have revealed significantly decreased levels of metastasis and disease recurrence (Liao et al., 2016). Lately, a newly synthesized triazolo–thiadiazoles (4-MMI) has been shown to successfully inhibit enzymatic heparanase activity and heparanase-mediated VEGF gene expression, restraining the ability of carcinoma cells to extravasate through the subendothelial basement membrane. It’s shown that 4-MMI yield a nearly fourfold inhibition of 4T1 breast carcinoma metastasis, comparable to the effect exerted by roneparstat (Barash et al., 2021).

### Other Promising Therapies

Methods that directly interfere with sheddase access to cleavages sites or stabilize the SDC-1 core protein into a confirmation less prone to proteolysis may also be good strategies to control SDC-1 shedding and subsequent tumor progression. The shedding of SDC-1 is modulated by the intracellular binding of the small GTPase Rab5 to the SDC-1 CD; Rab5 dissociation from the SDC-1 CD results in SDC-1 shedding and prevention of syndecan-Rab5 complex dissociation is thus considered to be another promising approach (Hayashida et al., 2008).

Creation of anti-tumor HS fragments can be accomplished by degrading HS *in vitro* with a bacterial enzyme and then administering the resultant fragments to tumor-bearing mice. This method has successfully stopped tumorigenesis in animal models of melanoma and myeloma (Liu et al., 2002; Yang et al., 2007). Although targeting proteoglycan remains more challenging than targeting heparanase, therapeutic potential has nevertheless been demonstrated with the former strategy. Hydrophobic aglycones can be used to disrupt the normal assembly of glycosaminoglycan chains on proteoglycan core proteins, stimulating the production of antiproliferative glycosaminoglycans and inhibiting proteoglycan synthesis. As a result, tumor progression and angiogenesis can potentially be prevented (Tsuzuki et al., 2010). Drugs targeting SDC-1 or SDC-1-related proteins are shown in Table 1.

**TABLE 1:** Drugs targeting SDC-1 or SDC-1-related proteins.

Target	Drug name	Chemical type	Mechanism of action	Effect	Cancer	References
SDC-1	Zoledronic acid	Resembles endogenous pyrophosphate	Disruption of SDC-1/integrins cross-talk	Inhibition of SDC-1 and ανβ3 integrin protein expression	Myeloma cells; breast cancer	Dedes et al. (2012)
	Nimesulide	Non-steroidal anti-inflammatory drug	Inhibition of SDC-1 expression	Inhibition of cell migration	Primary effusion lymphoma	George Paul et al. (2011)
	Indatuximab ravtansine (BT062-DM4)	Antibody-drug conjugate to cytotoxic agent (DM4)	Cytotoxic action in the target cell	Specific SDC-1^+^ cell death; inhibition of tumor cell growth and proliferation	Multiple myeloma	Schönfeld et al. (2017), Jagannath et al. (2019)
	OC-46F2	Recombinant antibody	Inhibition of SDC-1/VEGFR2 interaction	Inhibition of vascular maturation and tumor growth	Malignant melanoma and ovarian cancer	Orecchia et al. (2013)
	(CDB-HepII) polypeptide	Polypeptide	Reduction in αVβ3 integrin and SDC-1, interaction	Inhibition of pulmonary metastasis	Melanoma	Gong et al. (2008)
	Synstatin	Peptide	Competition with SDC-1 to bind receptors	Blockage of the SDC-1 core protein active site; suppression of the EGFR/α6β4 integrin complex; inhibition of angiogenesis	Multiple myeloma; mammary tumors	Beauvais et al. (2009), Rapraeger (2013), Rapraeger et al. (2013), Gao et al. (2021)
Shed SDC-1	Batimastat (BB-94)	Small molecule	MMP inhibition	Inhibition of SDC-1 shedding; prevention of tumor progression	Breast, ovarian, and colorectal cancer	Macaulay et al. (1999)
	NCS 405020	Small molecule	Inhibit the homodimerization of MT1-MMP	Reduction in SDC-1 shedding; suppression of tumor growth and invasion	Breast cancer	Remacle et al. (2012)
	All-trans retinoic acid (A-TRA)	Micronutrient	-	Reduction in SDC-1 shedding; inhibition of cancer invasion/metastasis	Lung	Ramya et al. (2012)
	Tranexamic acid	Synthetic lysine analogue	Inhibition of serine protease	Reduction in SDC-1 shedding; prevention of cancer metastasis	-	Diebel et al. (2018)
	Antithrombin III	Protein	-	Prevention of SDC-1 shedding; inhibition of metastasis	-	Lopez et al. (2020)
Heparan Sulfate	Heparin	Anticoagulant drug	Inhibition of heparanase activity	Hampering VEGF and FGF-2 activity, anti-angiogenesis	Myeloma	Casu et al. (2007)
	SST0001	Modified heparin	Inhibition of heparanase	Inhibition of HGF, VEGF, and anti-angiogenesis	Human pediatric sarcoma models	Cassinelli et al. (2013)
	M402	Modified heparin	Inhibition of heparanase	Anti-angiogenesis; inhibition of metastasis	Melanoma	Zhou et al. (2011), Kaur et al. (2021)
	9E8, H1023	Heparanase-neutralizing monoclonal antibodies	Neutralization of heparanase enzymatic activity	Inhibition of metastasis	Lymphoma	Weissmann et al. (2016)
	PG545	Sulfated synthetic tetrasaccharide	Inhibition of heparanase	Suppression of proliferation and migration	Ovarian cancer	Winterhoff et al. (2015)
	Suramin	Polysulfonated naphthylurea-based small molecule	Suppression of the activity of heparinase activity	Inhibition of caspase-3/8/9 activity; inhibition of FGF-2; suppression of both intrinsic and extrinsic apoptotic pathways	Hepatocellular carcinoma	Tayel et al. (2014)
	PI-88	Sulfated oligosaccharide	Inhibition of heparanase activity	Prevention of cancer recurrence and metastasis of cancer	Hepatocellular carcinoma	Liao et al. (2016)
	4-MMI	Triazolo–thiadiazole compounds	Inhibition of heparanase activity	Prevention of carcinoma cell invasion and metastasis; downregulation of VEGF expression	Glioma; breast cancer; myeloma	Barash et al. (2021)

## Role of MicroRNAs in SDC-1 Activity and Tumor Development

MicroRNA (miRNA), a class of short non-coding RNA, exerts its effects *via* the modulation of target gene expression at the post-transcriptional stage. Indeed, miRNA is capable of controlling heparanase/SDC-1/shed SDC-1 gene transcription in the setting of malignancy. The SDC-1 mRNA 3′-UTR is a direct target of miR-494, miR-515-5p and miR-302a. Notably, reduction in miR-494 levels results in enhanced SDC-1 shedding and angiogenesis in medulloblastoma cells, while miR-302a and miR-515-5p inhibit ovarian and bladder cancer cell growth correspondingly *via* the targeting of SDC-1 (Asuthkar et al., 2014; Guo et al., 2015; Cao et al., 2021). As such, miR-155-based artificial miRNA and lentiviral miR-30-based RNA can both target heparanase to suppress melanoma cell adhesion, migration and invasiveness *in vitro* (Liu et al., 2012; Liu et al., 2013). Syndecan binding protein (SDCBP), an adapter protein possessing PDZ domains, is known to interact with SDC-1 and be a target of both miR-135a-5p/miR-124-3p as well as miR-361-5p. *In vivo* studies have revealed that SDCBP silencing or miR-135a-5p/miR-124-3p and miR-361-5p overexpression resulted in a significant reduction in tumor growth among glioblastoma and gastric carcinoma cell-bearing animals (Lin et al., 2018; Qian et al., 2020). Other research has revealed a distinctly negative correlation between miR-135b-5p and SDCBP expression. Suppression of miR-135b-5p was found to be associated with facilitation of the EMT and migration of breast cancer cells. Thus, miR-135b-5p acts in the early prevention of breast cancer metastasis by targeting SDCBP (Pu et al., 2019). In addition, microRNA such as miR-1273a is also involved in SDC-1-associated chemoresistance. Expression of SDCBP is downregulated by miR-1273a. Overexpression of miR-1273a boosts cisplatin cytotoxicity while lower plasma exosome miR-1273a levels and higher plasma SDCBP levels are associated with poorer therapeutic results among patients who underwent platinum-based therapy and suffer advanced non-small-cell lung cancer (Zhao et al., 2020). Specific alterations in miRNA expression have been speculated to affect SDC-1 signaling and promote cancer progression. Future studies should focus on exploring the relationship between expression levels of specific microRNA and tumor phenotypes induced by SDC-1, whether microRNA expression levels can be reliably used as a prognostic indicator of SDC-1-related cancers, and possibly offer novel approaches for the targeting of SDC-1 to intervene in cancer pathogenesis. The targeting of SDC-1 or SDC-1-related proteins by miRNA is detailed in Table 2.

**TABLE 2:** The targeting of SDC-1 and SDC-1-related proteins by microRNA.

Target (gene)	Potential microRNA	Mechanism of action	Cancer	References
SDC-1	miR-494	Inhibition of SDC-1 shedding; inhibition of MMP-9, VEGF, and HIF1a; suppression of angiogenesis	Medulloblastoma	Asuthkar et al. (2014)
	miR-302a	Inhibition of the transition from G1 to S phases; inhibition of cell growth; increase in apoptosis	Ovarian cancer	Guo et al. (2015)
	miR-515-5p	Inhibition of cell proliferation, migration, invasion, and colony formation; increase in rate of cell apoptosis	Bladder cancer	Cao et al. (2021)
Heparanase	miR-155-based artificial miRNA	Suppression of melanoma cell adhesion, migration and invasiveness	Melanoma	Liu et al. (2012)
	Lentiviral miR-30-based RNA interference	Suppression of melanoma cell adhesion, migration and invasiveness	Melanoma	Liu et al. (2013)
SDCBP	miR-135a-5p/miR-124-3p	Inhibition of tumor growth; decrease in tumor size; prolong survival time	Glioblastoma	Lin et al. (2018)
	miR-361-5p	Inhibition of cell proliferation and tumor growth; increase in apoptosis	Gastric carcinoma	Qian et al. (2020)
	miR-135b-5p	Inhibition of the epithelial-mesenchymal transition; decrease in migration	Breast cancer	Pu et al. (2019)
	miR-1273a	Enhancement of cisplatin cytotoxicity; prevention of chemoresistance	Non-small cell lung cancer	Zhao et al. (2020)

## Discussion

Pathologic SDC-1 expression interferes with complex molecular signals and impact tumor grade, invasiveness and prognosis. Available data have supported cell-surface binding, shedding and nuclear localization of SDC-1 to contribute to cancer progression, although study of such phenomena has remained unsystematic. Levels of SDC-1 expression have, however, been recognized as a prognostic marker in solid and hematologic cancers. As molecular biology and detection technology continues to advance, studies are increasingly focusing on the effects of cellular SDC-1 localization on cancer prognosis and disease phenotype. Shed SDC-1 promotes binding between growth factors and their receptors, or in other cases acts as decoy receptors. The intranuclear presence of SDC-1 activates gene transcription and influences various physiological activities. Awareness of the different effects exerted by varying cellular SDC-1 localization, and especially of the role shed SDC-1 plays in aggressive cancer phenotypes, is of vital importance in furthering understanding of mechanisms relevant to cell growth and proliferation, angiogenesis, metastasis and chemoresistance. Importantly, SDC-1 is a potentially attractive molecular target that can guide individualized cancer diagnosis and treatment.

Although available data is interesting, drawbacks in knowledge remain. Based on prior studies, specific associations between SDC-1 expression in different cells and cancer occurrence in different tissues cannot be made. Likewise, correlations between tumor characteristics, SDC-1 localization and prognostic significance in the setting of different cancers warrant further research. As precision medicine continues to emerge, cancer treatment regimens increasingly require both cancer- and patient-specific individualization.

Further study of the roles SDC-1 plays in cancer metastasis and drug resistance warrants: a) exploration of which pathological SDC-1 expression responds to cancer progression more rapidly by analysis of cancer tissue, tissue adjacent to the tumor and bodily fluids; b) conduction of cohort studies evaluating the association between specific cancers and SDC-1-related molecular or genetic expression; c) determination of dysregulation of which domain (i.e., CD, TMD or ED) is decisive for cancer phenotype formation among various malignancies; d) quantification of SDC-1 expression in different domains in the setting of various disease (i.e., TNM) stages; and e) further exploration whether the pathological expression of SDC-1 is associated with a specific predisposition towards cancer metastasis.

In conclusion, SDC-1 is a promising biomarker that can contribute to cancer diagnosis and prognosis. This protein is critical in the future individualization of targeted therapies for patients suffering poorly prognostic malignancies with high relapse rates.
