# Erratum: The role and therapeutic value of syndecan-1 in cancer metastasis and drug resistance

**DOI:** 10.3389/fcell.2022.1074502

**Published:** 2022-11-03

**Authors:** Frontiers Production Office

**Affiliations:** Frontiers Media SA, Lausanne, Switzerland

**Keywords:** syndecan-1, metastasis, drug resistance, therapy, cancer

Due to a publishing error, the symbol which displays first co-authorship for authors Sen Guo, XinYi Wu, and Ting Lei was missing.

The publisher apologizes for the mistake. The original article has been updated.

